# Delineation of amplification, hybridization and location effects in microarray data yields better-quality normalization

**DOI:** 10.1186/1471-2105-11-156

**Published:** 2010-03-26

**Authors:** Marc Hulsman, Anouk Mentink, Eugene P van Someren, Koen J Dechering, Jan de Boer, Marcel JT Reinders

**Affiliations:** 1 Delft Bioinformatics Lab, Delft University of Technology, Mekelweg 4, Delft, 2628 CD, The Netherlands; 2 Department of Tissue Regeneration, University of Twente, PO box 217, Enschede, 7500AE, The Netherlands; 3 Centre for Molecular and Biomolecular Informatics (CMBI), Radboud Universiteit Nijmegen, PO box 9101, Nijmegen, 6500HB, The Netherlands; 4 Department of Applied Biology, Radboud Universiteit Nijmegen, PO box 9101, Nijmegen, 6500HB, The Netherlands; 5 Department of Molecular Pharmacology, Merck Research Laboratories, PO Box 20, Oss, 5340BH, The Netherlands; 6 Physiological Genomics Group, BU Biosciences, TNO Quality of Life, PO Box 360, Zeist, 3700AJ, The Netherlands

## Abstract

**Background:**

Oligonucleotide arrays have become one of the most widely used high-throughput tools in biology. Due to their sensitivity to experimental conditions, normalization is a crucial step when comparing measurements from these arrays. Normalization is, however, far from a solved problem. Frequently, we encounter datasets with significant technical effects that currently available methods are not able to correct.

**Results:**

We show that by a careful decomposition of probe specific amplification, hybridization and array location effects, a normalization can be performed that allows for a much improved analysis of these data. Identification of the technical sources of variation between arrays has allowed us to build statistical models that are used to estimate how the signal of individual probes is affected, based on their properties. This enables a model-based normalization that is probe-specific, in contrast with the signal intensity distribution normalization performed by many current methods. Next to this, we propose a novel way of handling background correction, enabling the use of background information to weight probes during summarization. Testing of the proposed method shows a much improved detection of differentially expressed genes over earlier proposed methods, even when tested on (experimentally tightly controlled and replicated) spike-in datasets.

**Conclusions:**

When a limited number of arrays are available, or when arrays are run in different batches, technical effects have a large influence on the measured expression of genes. We show that a detailed modelling and correction of these technical effects allows for an improved analysis in these situations.

## Background

Most applications of oligonucleotide arrays, such as finding differential expressed genes or network reverse engineering, involve the comparison of different arrays. Since array measurements are highly sensitive to the experimental conditions, comparison of arrays can be problematic. This is especially the case when experiments have been performed in different batches or experiments. Several normalization methods have been developed to handle this problem (e.g. MAS 5.0 [1], VSN [2], RMA [3], PDNN [4], PLIER [5], GCRMA [6]).

In this work we focus on differences between arrays caused by amplification, hybridization and location-based effects. Often used normalization methods such as RMA do not take into account these significant technical effects, while methods such as PDNN and GC-RMA only take into account the hybridization effect (although in a different way than we propose). We introduce a new normalization method that takes into account all these effects and improves performance over the existing methods. Although this study focuses on Affymetrix GeneChips, the resulting method can also be applied to other oligonucleotide platforms.

### Technical effects

**Figure 1:**
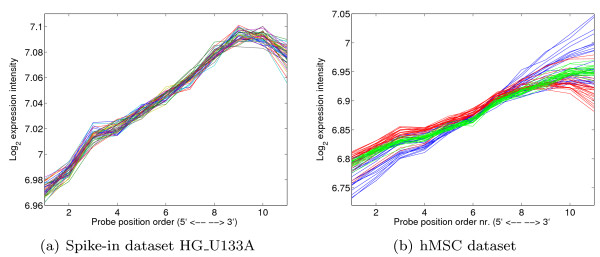
**Dependance of the measured signal w.r.t. probe position**. Probes have been ordered on their position along the mRNA molecule. Most probe sets consist of 11 probes. In these figures, we show the median expression profile (after quantile-quantile normalization) for the first probe in all probe sets, the second probe in all probe sets, and so on for 11 probes (for probe sets with a different number of probes we use interpolation). Each line in both plots indicates a separate microarray. The different colors in a) represent different arrays, and in b) represent different batches. For a description of the datasets see the Methods section.

The Affymetrix platform uses arrays with short 25-nucleotide probes placed on them. To measure mRNA expression, transcripts are amplified, fragmented and labeled, after which they are placed on the array to hybridize with the probes. After washing, the amount of hybridized RNA can be measured per probe. The first step that can easily be influenced by experimental conditions is the amplification. In this process, a primer is used to bind to the poly-A tail of a transcript, after which T7 RNA polymerase uses this primer to start the creation of new (complementary) copies of the transcript. We found that the array signal shows a negative bias for probes closer to the 5' end of the transcript (Figure 1). This effect has been identified earlier, and is part of the quality control measures in MAS 5.0 and the affy package [7]. Some authors have suggested that the 5' end negative bias is caused by 5' end RNA degradation. We found that an incomplete amplification (i.e. copies only cover part of the transcript) better explains the data (Additional file 1, Section S5). Currently, such an effect is not taken into account by any normalization methods that we know of.

**Figure 2:**
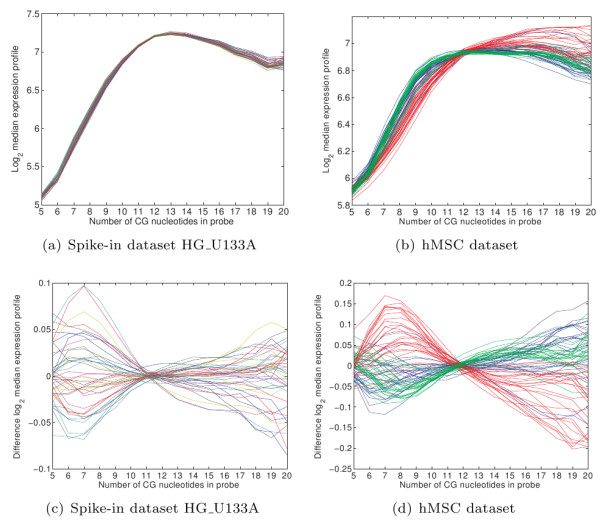
**Dependance of the measured signal w.r.t. probe sequence**. Probes consist of 25 nucleotides. For probes with the same number of C, G nucleotides we calculated the median signal for each array (a, b) or median of the difference w.r.t. the median signal over all arrays (c, d) (both after quantile-quantile normalization). The different colors in a) represent different arrays, and in b) represent different batches. For a description of the datasets see the Methods section.

The second step that can be influenced is the hybridization and washing of the fragmented transcripts (Figure 2). It is well known that these effects are generally dependent on the sequence [4,8-11]. In several current normalization methods (GCRMA, PDNN), the probe sequence information is used to remove signal bias and/or correct for the rate of binding of transcripts to the probe. In contrast this work focuses on using the hybridization model to reduce signal *variance*.

**Figure 3:**
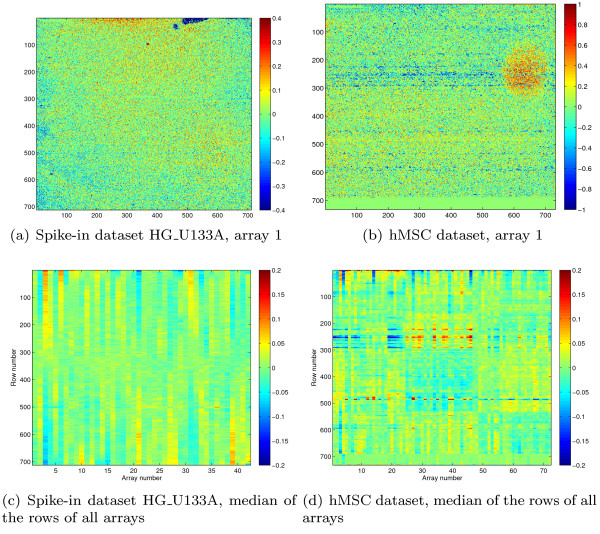
**Dependance of the measured signal w.r.t. probe array location**. (a, b) The image shows for each probe the log_2 _signal difference w.r.t to the median array (after quantile-quantile normalization), mapped to the location of the probe on the array. The value of MM-probes (which are located next to their corresponding PM probe) are set to the residual of their corresponding PM probe. (c, d) These images give an overview of the global array effect over all arrays. Each array is represented by a column, which is obtained by taking for each row in that array the median of values as shown in (a, b). For a description of the datasets see the Methods section.

The third technical effect is based on the location of the probe on the array. Some parts of the array show blemishes which reduce or increase the signal. We also find that large parts of the arrays can be affected between batches (Figure 3). We estimate this effect for every probe and remove it from the signal. A more simple 16-block grid based method is part of the MAS 5.0 method, while [12,13] divide the array in subarrays and apply normalization on each subarray separately.

### Background removal after normalization phase

Microarray pre-processing methods often have a background signal subtraction phase (bias reduction, i.e. removing signal consisting of optical background and signal due to non-specific binding), followed by a normalization phase (variance reduction) and summarization of the probe sets. The primary goal of the first phase is to improve accuracy (i.e. match the true signal level more closely), while the second phase is performed to improve precision (i.e. consistency of measurements over different arrays). Current pre-processing methods differ mostly in the method of bias reduction. For example, MAS 5.0 uses mismatch probes to estimate the background, RMA uses a general background distribution and GCRMA a sequence-based model. For normalization, often general distribution-correcting methods such as quantile normalization [14] or loess normalization [15] are used. One can perform these methods using a single reference array, or use multiple reference arrays as is done in the PTR method [16].

Our first attempt at removing technical effects during the bias reduction phase did show that, although one can improve accuracy, it is hard to not simultaneously decrease precision. The reason for this is that the estimated correction factors (biases) can be large and are estimated for each array separately with models that are simplifications of reality. To solve this, we perform the background subtraction phase after the normalization phase. Consequently, within the normalization phase, *differences *in technical effects between arrays are corrected. For example, in the case of sequence effects we perform now variance normalization (Figures 2c, d) instead of bias removal (Figures 2a, b). As we normalize not only the true signal but also the background signal, this allows us to use the same background estimate for all arrays during the background removal phase. This way we can still improve accuracy, while simultaneously preventing the reduction of precision.

### Background removal within summarization phase

In general it is true that in cases that the measured fold change between arrays is low one is less certain that there is actually a 'real' fold change as opposed to situations where there is a large fold change. An important factor influencing the measured fold change is (again) the background signal. Probes with a relatively large background signal will generally have a smaller fold change, if the fold change is calculated over the whole signal (background + foreground).

Currently, most methods remove the background before the summarization of the probe sets. Although this reduces signal and fold change bias, it also obscures the 'real' fold change. That is, the fold changes of probes with a large background signal will be blown up more than those of other probes. In fact, probes which measure only background signal for some arrays could get infinite fold changes if this was not prevented by limiting the amount of background subtracted. This has a major impact on the summarization of probe sets, as such probes become more important than they should.

One could choose to perform no background subtraction, preventing the dominance of high-background probes, at the cost of increased bias. But, even then probes that measure mostly background signal influence the summarization outcome. Therefore, in our approach, we have moved the background removal not only after the normalization phase, but into the summarization phase. This allows us to model the importance of the probes during summarization according to the amount of 'true' signal they measure.

## Results and Discussion

### Algorithm overview

**Figure 4:**
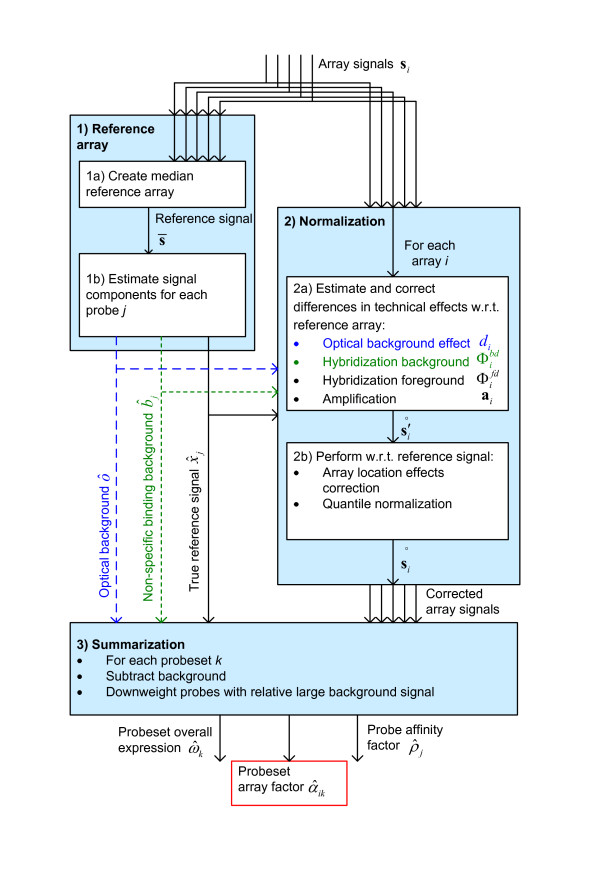
**Method overview**. High level overview of the steps from measured signal to summarization output.

To perform normalization, one has to determine what differences between arrays are caused by technical effects. However, performing pairwise comparisons of the arrays would lead to a quadratic number of comparisons, which does not scale for a large number of arrays. For this reason we construct a reference array (Figure 4, step 1a) based on the median of the signal:  = median_*i*_(*s*_*ij*_) (with *s*_*ij *_the measured signal of the *j*'th probe on the *i*'th array), and compare all the arrays to this reference array.

An often used model (e.g. [6]) for background removal is to split the signal in components according to *S *= *O *+ *B *+ *X*, where S is the measured signal, *O *represents the optical background, B is the background signal caused by non-specific binding, and *X *is the 'true' signal (in which we are interested). Here, we do not only use this model for background removal, but also for normalization, as some technical effects only affect a subset of these components. For example, amplification only affects *X*, while location-based effects appear to influence mostly *B *+ *X *(Additional file 1, Section S6). Our method works by first estimating values for these components for the reference array (Figure 4, step 1b), obtaining(1)

with  being the reference optical background, non-specific binding background and 'true' signal (the reference components). Then, in the normalization phase, we first normalize for the optical, hybridization and amplification effects (Figure 4, step 2a). These effects modify the components of the reference array signal into the measured signal in the following way:(2)

Here, *d*_*i *_is the optical effect difference, *τ *is the hybridization model and *κ *the amplification model. The parameters of these models (*d*_*i*_,, and **a**_*i *_respectively) are fitted by minimizing the difference between *s*_*ij *_(the measured signal of array *i*) and  (i.e. the reference array corrected for the differences caused by the technical effects for array *i*):(3)

This minimization is done robustly using a Huber M-estimator [17], for every array *i *separately. Note that the difference is in log scale, as the technical effects we are removing are mostly multiplicative effects. We obtain  by using the optimized parameters in Equation 2. This enables us to get an estimate of the technical effect difference for an array *i *w.r.t. the median pseudo-array using *t*_*ij *_=  - . We substract this technical effect difference from the measured signal *s*_*ij *_for array *i*, to obtain the corrected signal for these effects, i.e.:(4)

Using this signal, we perform in the next step array location correction (*L*(.)) and distribution correction using quantile normalization (*Q*(.)) (Figure 4, step 2b). The reason to do these corrections separately is that these effects cannot be effciently estimated using the approach of equation 3. As technical effect estimations can be dependent on each other, one could choose to perform the normalization steps in an iterative fashion (i.e. repeatedly applying each step until convergence). We found, however, that repeating the first part (step 2a) of the normalization is unnecessary, as it did not add noticeable performance. For the second part (step 2b), one could first perform array location correction and then quantile normalization, or the other way around. However, as quantile normalization is a fast and idempotent procedure, we perform quantile normalization both before and after array location correction, i.e. . Also in this case we did not find noticeable performance advantages over a non-iterative solution.

In the next subsections we discuss how to estimate the reference signal components (Equation 1), as well as the hybridization and amplification model (Equation 2). Then we discuss the array location correction performed during step 2b, and the final step in the algorithm, the summarization and background removal (Figure 4, step 3).

#### Estimating signal components

To divide the reference array signal  in its components (Equation 1) we first estimate  and . To estimate the reference optical background and the reference background due to non-specific binding, we use respectively a scalar and a sequence-based hybridization model (the same model as was also used to model the hybridization differences in Equation 2). Using these, we try to explain as much of the reference signal as possible. Although we could use  as target signal, we found that using min_*i*_(*s*_*ij*_) performs slightly better, presumably because it is a closer estimate of the 'only-background' signal. The estimate then becomes:(5)

where *τ*_*j *_represents the hybridization model for probe *j*, *ϕ*^*bg *^represents the hybridization model parameters, and *w*_*j *_is a weight assigned to each probe indicating whether the probe violates the background model and is calculated according to:(6)

The reason for this non-symmetric least-square weighting is to prevent an estimated background that is larger than the measured signal. In this work we use *η *= 10. The model is estimated robustly using a (iteratively reweighted least square) Huber M-estimator. Then we determine *B *and *X*, in such a way that *S *≥ *B *+ *O *using(7)

and(8)

#### Hybridization model

The hybridization model *τ*_*j *_is used three times: to estimate the reference background signal  (Equation 5) as well as to estimate the background and foreground hybridization *differences *between arrays (Equation 2). The separate background and foreground hybridization difference model represents the notion that the non-specific hybridization process differs from the hybridization process of the targeted sequence [4].

The hybridization model is based on the probe sequence. Earlier studies have indicated that the position of nucleotides on the probe are important [4,8]. Furthermore, [4] as well as [10] suggested to fit dinucleotide binding strength, based on hybridization experiments in earlier studies. Each dinucleotide is presumed to have a multiplicative effect on the signal. As additive models are easier to handle, we use the following transformation: . This allows us to estimate the effects as additive components of .  is modelled according to two parts. One part models the influene of nucleotide pairs in the probe sequence (part 1, *τ*_(1),*j*_). The other part models the influence of the number of certain nucleotides present in the probe sequence (part 2, *τ*_(2),*j*_).

Let *ζ*_*j *_represent the (length 25) sequence of probe *j*, and *ζ*_*j*,*m *_∈ ('A', 'C', 'G', 'T') the *m*'th nucleotide on it. Although we could fit a model where we determine an influence factor for every nucleotide pair for every position (i.e. , where *ζ*_*j*,(*m*,*m*+1) _is defined as {*ζ*_*j*,*m*_,*ζ*_*j*,*m*+1_}), this would lead to a large amount of variables. As these influence factors vary smoothly with probe position, it is possible to estimate the position effect with fewer parameters. To this end, we used B-splines (see Additional file 1, Section S4), i.e.(9)

where *ϕ*_*p*,*q *_is a knot weight *q *for nucleotide pair *p*, and *B*_*p*,*q*_(*m*) is the corresponding B-spline basis function factor for position *m *on the probe. Furthermore, *I*((*ζ*_*j*,(*m*,*m*+1) _= *p*) is an indicator function determining if probe *j *has nucleotide pair *p *on positions (*m*, *m *+ 1).

This model assumes that all contributed binding affinities contribute linearly to the sequence effect in log-space. In reality we did not find this to be the case. For example, probes with relatively many adjoining C, G nucleotides reported consistently a higher signal than would be expected from the linear model. For this reason, we added factors to the model that depend on the number of certain nucleotides within a probe. Let *c*(*N*, *ζ*_*j*,(*v*,*w*)_) = ∑_*n*∈*N*_|*ζ*_*j*,*x*_| *ζ*_*j*,*x *_= *n*, *v *≤ *x *≤ *w*| represent the number of certain nucleotides *N *in a (part of) the probe sequence *ζ*_*j*,(*v*,*w*)_, then the second part of the sequence model is:(10)

where *r *is a sequence range parameter with elements from *R *= {(1, 25), (1, 13), (14, 25)}, *V *∈ {'A', ...'T', {'A','C'}, ...} contains the nucleotide sets that were counted in each probe, *B*_*v*,*r*,*q*_(.) is the B-spline basis function factor with as parameter the number of nucleotides that fall within range *r *and are in set *v*, and *ϕ*_*v*,*r*,*q *_is the corresponding weight for knot *q*. We use the different sequence ranges *r *to better model the non-specific background binding which often only hybridizes with a part of the probe sequence. The final model is then the summation of the two introduced parts:(11)

Experimentally, we found that *Q *= 5 knots with degree 3 (cubic B-splines) are able to fit the signal well. Our results indicate that there is enough data to estimate the 230 parameters within ϕ without significant overfitting. This is to be expected as there are often more than 200,000 probes on a microarray.

#### Amplification effect correction

Our experiments (Additional file 1, Section S5) indicate that the lower expression of 5' end probes is best explained by incomplete amplification of the probes. Copies are made starting from the 3' end of the transcript, using a primer attached to the poly-A tail. However, not every copy will be complete, causing a 3' end bias in the signal we measure. This suggests that differences in this effect between arrays could be modeled using the distance between a probe and the poly A-tail. As it is somewhat complicated to determine the location of this poly-A tail w.r.t. to the probe set (due to splicing), and most probe sets are located close to the poly-A tail, we make the simplification that we consider the amplification differences to start at the 3' end of the probe set. We found that a simple (linear) probe location model, *p*_*j*_*a*_*i*_, where *p*_*j *_is the distance of a probe to the 3' end of the probe set and *a*_*i *_an amplification difference parameter, did not remove most of the amplification effect. We suspected that the amplification effect is actually sequence-dependent, and changed our model to include these effects:(12)

where probe-specific vector **p**_*j *_contains for each dinucleotide the number of occurences between the middle of probe *j *and the 3' end of its probe set, and the array-specific vector **a**_*i *_contains the parameters for each dinucleotide determining its role in amplification differences. An F-test shows that this model significantly improves the amount of amplification effect our model is able to fit over the location-only linear model (Additional file 1, Section S5).

#### Array location effects

Estimating the (per-probe) array location effects within the model itself would lead to an unrealistic large number of parameters. For that reason, we estimate the array location effects separately. That is, for a given input signal *x*_*ij*_, we calculate an output signal *y*_*ij *_= *L*(*x*_*ij*_) corrected for location effects. In the 'Algorithm overview' section it is described how this function is used in the second normalization step. Although we cannot exclude that location effects could affect the optical background signal, we found that normally only the hybridization signal (both background and foreground) is affected (see Additional file 1, Section S6). Furthermore, even if there are location effects in the optical background they will not have a significant impact on the signal of expressed genes. For this reason, we estimate the location effect only for the hybridization signal. Assuming that the input signal has already been normalized for optical background (as we do in Figure 4, step 2a), we can use a common optical background estimate .

To perform the location correction, we determine the difference between the median signal over all arrays and the input signal, for each array *i*. The difference between these two is calculated after optical background subtraction, using:(13)

We use log scaling as the location effects affect the signal multiplicatively. Next, all calculated residues ϵ_*ij *_are mapped to the arrays on the location of their corresponding probe *j *(see Figure 3). If there is a location effect in a certain region of an array this will show as residues with a negative or positive bias. To robustly estimate this effect, we calculate for each probe on each array the median of the residues in the neighborhood. However, not every place on the array contains a probe, furthermore we do not use the mismatch probes. To handle the empty probe positions as well as probes near the border of the array, we choose to use a fixed size median box filter of 9 × 9, which takes into account only the valid probes under it. This means that the number of probes used by the filter depends on its position. The result of this filter are location effect values *λ*_*ij *_= *median_box_filter*(**ϵ**_*i*_, *j*) for each probe on each array. To obtain the corrected signal we calculate .

#### Summarization

The normalized signal  is summarized for each probe set *P*_*k *_(1 ≤ *k *≤ *K*). Instead of using median polish (RMA) or the Tukey bi-weight (MAS5) we apply here a Huber M-estimator which was found to lead to better results [18]. Also, it enables us to use a more flexible model, which uses the estimation of the background signal, such that probes with a high background signal w.r.t. to the true signal obtain a lower weight. Similar to other summarization methods, we estimate a mean probe set expression *ω*_*k*_, a probe set array factor *α*_*ik *_containing fold changes w.r.t. to *ω*_*k*_, and a probe affinity factor *ρ*_*j*_. These factors are estimated using the following model:(14)

To make the model identifiable, we use the constraints ∑_*i*_*α*_*ik *_= 0 ∀*k *and ∑_*j*_*ρ*_*j *_= 0. When the mean true signal level for a probe is low (i.e. ), the array effect *α*_*ik *_will have less influence on the magnitude of the residual signal. Due to this, the probe will have less influence on the final summarized signal.

A second modification we make is the removal of outlier probes. This has been suggested before in [18]. If the Huber M-estimator (see Additional file 1, Section S3) assigns a low weight to the measured values of a probe (i.e. < 0.9) for more than one-third of the arrays we remove the probe entirely as its quality for the other arrays is also questionable. However, we make certain that we keep always more than 5 probes within a probe set.

The fold change values *α*_*ik *_are used for further analyses. These fold changes are corrected for bias as the background signal has been removed. Using bias-corrected fold changes can be advantageous when performing for example network reverse engineering. However, as discussed in the introduction, one looses information on the reliability of the fold change. This is one of the reasons that methods such as MAS 5.0 and PLIER (which perform a strong background correction, removing most of the bias) do not perform that well when used with differential expression detection algorithms based on the magnitude of the fold change. To prevent this, we have added an option to backscale the fold changes using . This is the final output of the algorithm and is used in the subsequent analyses.

### Differentially expressed gene detection performance

To determine the detection performance on differentially expressed genes we used two spike-in datasets, one using the HG_U95A platform and one using the HG_U133A platform (a description of the used datasets can be found in the Methods section). We compared (Table 1) the ROC50 score for our method as well as several other popular normalization methods. The main result is that for both datasets the proposed method (referred to as Robust Difference Normalization (RDN)) obtains the highest performance (i.e. respectively an ROC50 score of 0.89 and 0.80). If we look at the fold-change ranking, the next-best method is RMA for the HG_U133A dataset and GCRMA for the HG_U95A dataset. However, both methods do not perform consistently well over both datasets.

**Table 1:** An overview of the results for the two spike-in experiments

(ROC50 scores)	HG_U95A			HG_U133A		
Method name	fold chg. single repl.	SAM all	fold chg. all	fold chg. single repl.	SAM all	fold chg. all
MAS 5.0	0.00	0.25	0.01	0.01	0.45	0.02

PLIER	0.00	0.49	0.01	0.04	0.61	0.10

VSN	0.62	0.42	0.77	0.65	0.32	0.70

PDNN	0.63	0.45	0.73	0.59	0.70	0.71

RMA_NBG	0.64	0.53	0.78	0.69	0.66	0.77

RMA	0.47	0.27	0.75	0.68	0.70	0.77

GCRMA	0.71	**0.68**	0.84	0.65	0.57	0.72

RDN	**0.80**	0.66 (**0.76**)	**0.89**	**0.75**	**0.77**	**0.80**

RDN [qq]	0.67	0.57	0.80	0.71	0.65	0.77

RDN [qq, loc]	0.70	0.65	0.83	0.72	0.69	0.78

RDN [qq, hyb]	0.76	0.74	0.87	0.75	0.75	0.80

RDN [qq, hyb, amp]	0.76	0.73	0.87	0.75	0.75	0.80

RDN [qq, hyb, loc]	0.80	0.68	0.88	0.76	0.77	0.80

See Additional file 1, Table S1 for a description of the methods. Both fold change and the SAM test statistic were used to determine ROC50 scores (see Methods section). The 'single repl.' score is determined by applying the normalization and scoring methods to each of the three replicate array-sets individually and averaging the results, while the 'all' score is obtained by using all replicates simultaneously. The lower 5 rows describe versions of RDN which use a subset of quantile-quantile normalization ('qq'), array location ('loc'), hybridization ('hyb') and amplification ('amp') correction to determine their individual influence.

Every spike-in experiment was performed three times. We compared the performance both by using the methods on single replicates (and averaging the performance over the three replicates) as well as by applying the methods on the complete dataset simultaneously. As expected, the performance gain when using each replicate separately is larger, as replication is performed to reduce the effect of unwanted differences between arrays. However, even with replication our method outperforms other methods. In this context, it is also interesting to look at the SAM test statistic score. Many studies do not have multiple replicates for each sample (as is done here), but use multiple samples per class. When comparing such classes, often the SAM(-related) test statistic score is used, which takes into account variability within a class to determine the ranking. Reducing variability caused by technical effects can improve its results. Here we see that RDN performs better than many of the other normalization methods. The slightly higher SAM score of GCRMA for the HG_U95A dataset can be attributed partially to its use of a low probe expression cutoff, thereby removing probe sets with very low expression from consideration. If we apply a similar cut-off to the same amount of probes as in GCRMA, we obtain a better SAM-score of 0.76. To determine the performance effect of individual components of RDN, we ran our method with certain normalization steps disabled (Table 1). When performing only a standard quantile normalization in combination with the new summarization procedure, RDN already outperforms most of the other methods. Enabling correction for location-specific effects as well as hybridization effects further increases the performance. Amplification correction on the other hand does not significantly add to the performance for these experiments. This is as expected, as there are no significant differences in amplification between the arrays (see Figure 1a, the lines are more or less parallel), presumably because the amplification was done once for all arrays, which in practice is not realistic for most experiments.

### Differential gene finding - hMSC dataset

The hMSC dataset has been measured in three batches, which each show differences in technical effects (see Figures 1b, 2d, 3d). As is the case for most biological datasets, we do not know the exact true outcomes for the hMSC dataset. For this reason, we can only use the internal consistency of the dataset after normalization as a measure. Besides using the difference between replicate array pairs (for which RDN outperforms the other methods, see Additional file 1, Section S1), we also looked at differentially expressed genes. We used the binary labels for gender and sample bone location for this purpose. The normalization methods are applied on the full dataset. Then we determine for each batch separately the list of top genes w.r.t. to the class labels using SAM, and determine the overlap between the lists of top genes from the different batches. For this, we calculate an overlap score. Let *T*_*b*,*m *_be the list of *m *most significant genes for batch *b *w.r.t. to a certain set of class labels, then a score for each gene, *g*, can be calculated that expresses how often it is present in one of the lists of *m *most significant genes for each of the different batches:(15)

where |*B*| represents the number of batches, and *I *is an indicator function with value 1 if *g *is part of the top *m *genes of a batch, otherwise 0. After determining the gene scores, we determine the total overlap score of all genes, *o*(*m*), by summing the individual gene scores. Similar to an AUC score, the final overlap score is calculated by determining the area under the *o*(*m*) function with 1 ≤ *m *≤ *M*, while normalizing for *M*, i.e:(16)

**Figure 5:**
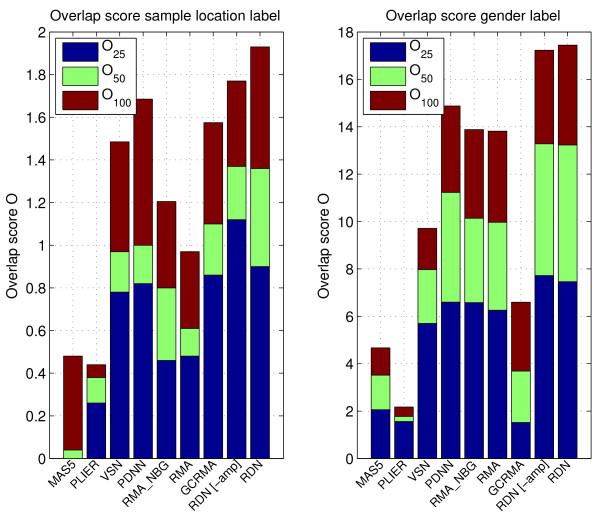
**Overlap between top gene lists**. Final overlap score, *O*_*m*_, between top gene lists obtained by applying SAM to the three different batches of the hMSC dataset. (see also Equation 16).

We report this score for various *M *in Figure 5. Despite the large number of samples (which reduces the need for extra normalization), RDN performs on average better than other normalization methods. The next best method (PDNN) is also a method that corrects for sequence effects, confirming that this has a positive effect. We determined the influence of amplification correction by disabling it. Here results are less conclusive. Although we see an improvement for the location label, this is not the case for the gender label.

### Signal bias and background estimation

**Figure 6:**
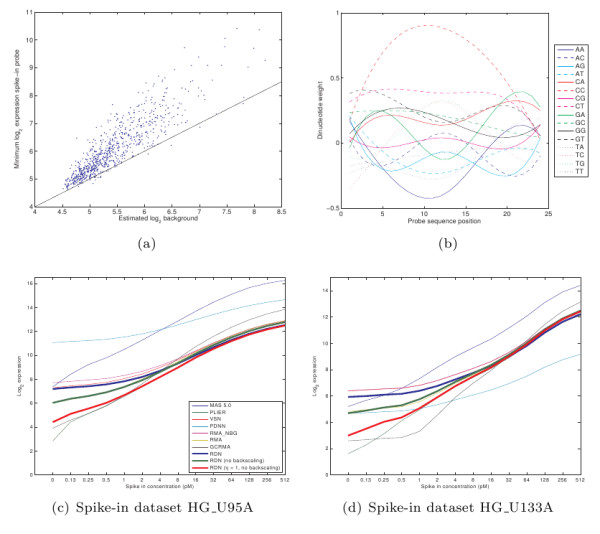
**Background and signal accuracy**. (a) Comparison between estimated background signal (*b*_*j*_) and measured spike-in probe signal (*s*_*j*_) on the HG_U133A platform, for spike-in concentrations of 0. Results for other platforms are similar. (b) Estimated dinucleotide weight with respect to probe position on the HG U133A platform. (c, d) Calculated average expression levels for the spike-in probe sets at different spike-in concentrations, showing the bias in signal levels. The slope of the line represents the scale of the fold changes (for *r*^2 ^and slope values see Tables S6 and S7 in Additional file 1).

To remove signal bias, one needs a good background estimate. To compare the estimated background with the real background signal we used spike-in probes. For these probes we know that for a spike-in concentration of 0, we should only measure background signal [10]. Figure 6a shows the estimated and measured background signal. Although we only use a linear model, the estimator gives a relatively good approximation of the actual background signal. A perfect prediction of the background cannot be expected as our model only looks at dinucleotides and nucleotide counts, leaving out the effect of longer matching RNA sequences within the RNA sample. The underestimation of the background is caused by using *η *= 10 in Equation 6. We used this value to prevent overestimation of the background, which would remove true signal. In Figure 6b, we show the estimated dinucleotide weights w.r.t. to the probe position for the background model. As can be seen, 'CC' dinucleotides add to the background signal for a large part. Furthermore, we see that dinucleotides such as 'GG', 'GC' are preferentially bound to the nucleotides closest to the 5' end.

To correct for signal bias, the estimated background is removed from the signal during summarization (Equation 14). To determine the remaining signal bias after this procedure, we compared the corrected expression levels for the spike-in probe sets with their actual spike-in concentrations (Figures 6c, 6d). As these are log-log plots, unbiased signals should result in straight lines. In these plots, we see that PLIER and PDNN are the best performing method in terms of bias reduction (see also Tables S6 and S7 in Additional file 1, containing the *r*^2 ^measure as well as the slope for each of the methods). By default, RDN does not focus on bias correction as unbiased fold changes can negatively impact differential expression detection, as discussed in the introduction. In fact, after summarization it re-adds the background signal (backscaling). Due to this, RDN has approximately the same bias as RMA_NBG, a method which does not remove background signal. However, if one requires more accurate signals one can also use a different setting for RDN, i.e. no backscaling and no underestimation of background (*η *= 1). With this setting, RDN has a summed *r*^2 ^measure between PDNN and PLIER.

### Signal precision for low, medium and high expression spike-ins

Next to signal accuracy (improved by background removal), one can also look at signal precision (improved by normalization). However, one cannot use measures such as the standard deviation of a probe signal over multiple arrays. The reason for this is that background removal blows up the fold changes, especially of low expression probes. As each method uses different strategies for background removal, one can not directly compare such measures. A similar problem affects comparisons of signal precision using spike-in probes, where one determines the performance of the spike-in probe sets for different spike-in concentration ranges. For example, when testing low-expression spike-in probe sets, the performance for a method without background substraction will be affected by a relatively large number of high-expression false positives. For methods with background subtraction, it is the other way around.

**Figure 7:**
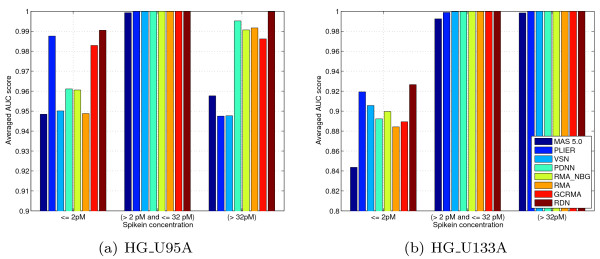
**Signal precision**. Signal precision for different spike-in concentration ranges. For each 2-fold spike-in concentration step the fold change and average expression of every spike-in probe set was determined over the arrays where the concentration step takes place. The fold change was compared to the fold change of 1000 probes closest in average expression (on the same arrays). The ranks are combined for a low, middle, and high spike-in concentration range, and an averaged AUC score is calculated.

Therefore, we report here on an experiment (Figure 7) in which we compared fold changes of spike-in probe sets only to the fold changes of probe sets that are closest in average expression. That way, the results are only negligibly affected by confounding effects due to signal/fold change bias. In this experiment, the highest precision is obtained by RDN. It is interesting, however, that PLIER performs quite good for low spike-in concentrations, indicating that mismatch probes can be useful for improving signal precision. However, for high expression spike-in probe sets (especially HG_U95A), PLIER does not perform that well, which is to be expected as mismatch probes are designed to report mainly background signal. Methods which also correct for foreground signal effects (RDN, PDNN) perform better here.

Another method to quantify precision is to rank replicate array pairs w.r.t. non-replicate array pairs based on the difference in expression between the arrays in the pair. Then one can compare the ranking using a method such as the AUC score. We did this on the hMSC dataset (Additional file 1, Section S1) and found that RDN outperforms all other methods.

**Figure 8:**
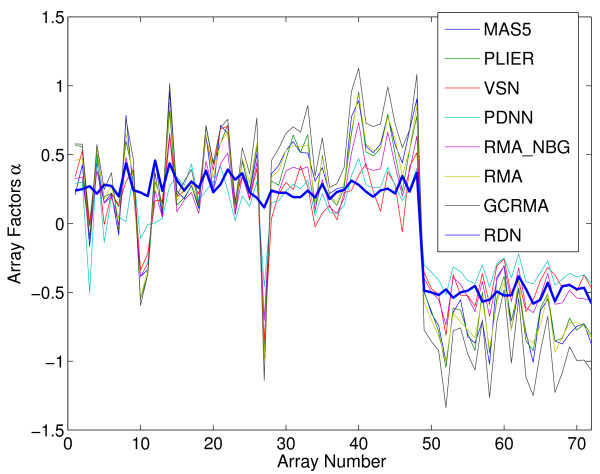
**bioC spike-in expression**. The gene bioC is spiked-in during the array experiments. This shows the concentration of the bioC gene in the hMSC dataset for multiple normalization methods, ordered by batch (batches 1,2,3, correspond to respectively array 1-24, 25-48, 49-72). The thick blue line is obtained by applying RDN.

A third way to determine precision is to look at genes that have been spiked-in into the hMSC dataset. We run here into the same problem as with using standard deviations, namely that different methods can have different fold change scales. Fortunately, we found that one gene (bioC) has been spiked-in at a different concentration for batch 3 of the hMSC dataset. This allows us to asses both the precision and fold change scale simultaneously, showing the strong reduction in technical variation obtained by using RDN compared to other methods (Figure 8).

### Inspection of dataset after normalization

**Figure 9:**
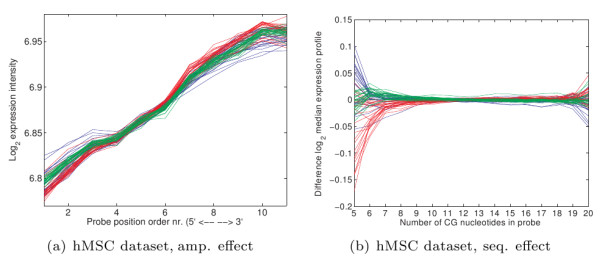
**Technical effects after RDN normalization**. Technical effects after RDN normalization (compare to figures 1 and 2 for the situation before normalization).

To validate the used models, we determined whether the technical effects are indeed removed after normalization. Results for the hMSC dataset (which has the largest differences in technical effects) are shown in Figure 9. Only for the less occuring types of probes, for example those with a GC content of 5, we see that there are still differences between the different arrays. This is caused by the limited amount of knots used in the B-splines, which however does protect the model from overfitting. We also see that a large part of the amplification difference has been removed, although there are still some outlier arrays. It is interesting to note that sequence correction also plays a role here. The drop in the signal on the 3' end of the probe set, which can be seen in Figures 9a and 1, is mostly caused by the relatively larger number of 'A' nucleotides close the 3' end of a gene. That is, the position on the gene and the sequence composition of the probes are not completely independent, suggesting that sequence and amplification effects should be estimated simultaneously as is done in our model. The array location normalization effectively removes trends in the array images (see Additional file 1, Figure S6), although locations where a large correction was necessary still have more variabilty in the residual signal, as is to be expected.

## Discussion

The results show that the performance improvements shown cannot be attributed to one component of the algorithm. Rather, it is a collection of improvements obtained by normalizing the different technical effects, removing the background after the normalization procedure, and, using background information in the summarization procedure.

Our method currently assumes that, on average, the expressions of genes remains similar accross multiple arrays, allowing us to identify the technical effects. This assumption is also used in other widely used normalization methods such as quantile normalization. The limitation of these type of methods is that they cannot be used when all genes change their expression simultaneously upwards or downwards. RDN requires more computation time than other normalization methods (10 minutes per array on a recent dual core 3 Ghz Intel processor). However, this amount of time is still small when compared to the time it takes to perform the microarray experiment. Furthermore, it is easy to parallelize the algorithm. Compared to the number of probes (> 200.000) our method uses a relatively limited numer of parameters. This prevents overfitting, so that only large scale effects affecting many probes simultaneously are removed. For platforms with even more probes one can choose to estimate the parameters with a subset of the probes. Currently, the amplification correction is based upon the assumption that probe sets are close to the poly-A tail of the probe set. This is not always the case. We found that especially probe sets further away from the poly-A tail can have large variations in signal for all probes, correlating with the amplification differences. This is not corrected by our current method. Given reliable distances towards the poly-A tail this could be added relatively easily.

The approach shown in this paper could also be useful for other oligonucleotide platforms. In this work we have mainly focused on gene expression. However, it could also be applied to ChIP-chip or tiling experiments.

## Conclusions

We have introduced a new normalization method that corrects for hybridization, amplification and array location effects that occur when measuring expression using oligonucleotide arrays. The proposed *Robust Difference Normalization *(RDN) corrects these technical effects by removing the differences in measured expression over different arrays instead of correcting for signal bias for each array. In this way, the proposed normalization procedure focuses on controlling the precision instead of the accuracy of the measured expression, which is more important for some applications (like, for example, detecting differentially expressed genes). We have shown that the proposed RDN method increases performance, even on experimentally tightly controlled and three times replicated spike-in data sets. The method will be most useful for studies consisting of few independent replicates or those showing large batch effects.

## Methods

### Data

Spike-in experiments have often been used for validation of normalization methods. We use two Latin Square experiments performed by Affymetrix on the HG-U95A and the HG-U133A platforms. These consist of respectively 59 and 42 arrays, and each measure 14 different spike-in gene groups. For each spike-in group, 14 different concentrations are measured. For the HG-U95A experiments these concentrations are 0, 0.25, 0.5 ... 1024 pmol, while for the HG-U133A experiment the concentrations are 0, 0.13, 0.25 ... 512 pmol. Fold changes between subsequent steps are equal to 2. For every measurement there are three replicates. The HG-U95A platform has extra replicates for some of the measurements. See Additional file 1, Section S2 for a description of which spike-in probe sets were used.

We also validated our method on a dataset measuring human mesenchymal stem cells (hMSCs). In this experiment, bone marrow aspirates were obtained from the acetabulum or iliac crest of patients undergoing hip replacement surgery, who had given written informed consent. Human mesenchymal stem cells (hMSCs) were isolated and proliferated as described previously in [19]. To analyze the gene expression profile we seeded hMSCs at 1000 cells/cm2 and upon reaching near confluence RNA was isolated using an RNeasy mini kit (Qiagen). Quality and quantity were analyzed by gel electrophoresis and spectrophotometrically. For 62 donors, RNA microarray experiments were performed on the HG-U133A 2.0 platform, in three different batches of respectively 21, 21 and 20 donors. Although experiments were performed at the same microarray facility, and with arrays from the same production batch, signficant technical differences were observed between the batches. Differences are likely due to the fact that the batches are performed at a different dates with several months in between. To validate our method, we used two different binary labelsets, namely the gender of the donors and the location (actabulum or iliac crest) from where the cells were obtained in the body.

### Spike-in performance

Using the Affymetrix spike-in datasets, we compared the performance of different normalization methods to detect differential expression between different spike-in concentrations. Each spike-in experiment contains 14 concentration groups, with each next group in the series having a 2-fold higher concentration. To make the comparison a more realistic approximation of reality, we only compared two subsequent concentration groups (limiting the comparison to 2-fold differences, including the step from 0 to the next lowest concentration). The resulting number of spike-in concentration pairs is 13 * |spike-ins|. We determine the fold-change or SAM test statistic [20] for the array pairs with the subsequent concentration groups. As is usually done, to determine the performance we calculate an area under the ROC curve (AUC score) using the spike-in pairs with 2-fold concentration difference as positive examples, and the non-spike-in pairs as negative examples. We limit the number of false positives to 50 for each array pair (also called ROC50 score), corresponding to regular practice (e.g. [21]) where one is only interested in results with a limited number of false positives.

### Quantile-quantile normalization

The quantile-quantile normalization is performed by mapping the signal distribution of different arrays to the distribution of the median array, after which the measurements from all arrays are sorted and replaced with values from the median array [14].

## Availability

The software is available as a Matlab Toolbox at http://bioinformatics.tudelft.nl/users/marc-hulsman

## Authors' contributions

MH developed the algorithm and drafted the manuscript. JB and ES participated in the design of the study. AL, KD and JB carried out/coordinated the experiments and helped with the biological interpretation. ES worked on the analysis of the data. MJT coordinated and participated in the development of the algorithm, and helped to draft the manusscript. All authors read and approved the manuscript.

## Supplementary Material

Additional file 1 **Supplementary information**. More details can be found in the supplementary information. Click here for file
