# Supplementary material for: Delineation of amplification, hybridization and location effects in microarray data yields better-quality normalization
Source: BMC Bioinformatics. 2010 Mar 26;11:156. doi: 10.1186/1471-2105-11-156 (PMC2857856; doi:10.1186/1471-2105-11-156)
Supplement: Additional file 1 — Supplementary information. More details can be found in the supplementary information. [file 1471-2105-11-156-S1.PDF]

Delineation of amplification, hybridization and location effects in microarray data yields better-quality normalization

M. Hulsman, A. Leusink, E.P van Someren,
K.J. Dechering, J. de Boer, M.J.T. Reinders

March 2, 2010

1 Biological variation versus technical variation

Normalization of the differences between microarrays should only remove technical variation and not biological variation. To show that the method does not just remove all signal differences (including biological variation), we used the replicate samples within the hMSC dataset. We compared the mean absolute difference in expression for replicate pairs against non-replicate pairs (which contain the biological variation). We do this by ordering the pairs on their mean absolute difference in expression. After normalization, replicate array-pairs should have on average the lowest mean absolute difference expression of all pairs. By labeling the array-pairs we can now calculate an Area Under the ROC-curve (AUC) score.

In the HMSc dataset there are three replicate measurements of the same mixture of 3 donors within both batch 1 and 2, as well as one further replicate in batch 3. Furthermore, 3 donors earlier measured in batch 1 or 2 were measured again in batch 3. As there are a total number of 72 arrays, this gives us 24 replicate pairs and 2532 non-replicate pairs. We also look at the score when only taking replicate-pairs within batches (6 replicate pairs, 2532 non-replicate pairs).

Results of the experiment can be found in Table 2, showing that the method we propose performs significantly better than existing methods. This is also the case when using only within-batch replicate pairs.

Method name	Background correction	Normalization method	Summarization method
MAS 5.0	Spatial effect and MM subtracted	scale	Tukey biweight
PLIER	PM - MM	quantile-quantile	Multiplicative model
VSN	Generalized log transform		median polish
PDNN	Combined model that subtracts sequence-based background and corrects probe sequence bias		
RMA	global background subtraction	quantile-quantile	median polish
RMA_NBG	-	quantile-quantile	median polish
GCRMA	sequence and mm-based background subtraction	quantile-quantile	median polish

Table 1: An overview of the earlier proposed methods tested in the experiments in the paper.

Method name	AUC	AUC (within-batch)
MAS 5.0	0.7707	0.9597
PLIER	0.7063	0.8962
VSN	0.6568	0.8176
PDNN	0.7383	0.8768
RMA_NBG	0.7672	0.9541
RMA	0.7397	0.9258
GCRMA	0.8540	0.9821
RDN	**0.9174**	**0.9980**
RDN [qq]	0.7624	0.9562
RDN [qq,loc]	0.7822	0.9667
RDN [qq,hyb]	0.9063	0.9972
RDN [qq,hyb,amp]	0.9143	0.9973
RDN [qq,hyb,loc]	0.9084	0.9979

Table 2: AUC score obtained by ordering replicate array-pairs and non-replicate array-pairs on mean absolute difference in expression.

2 Spike-in genes

We found that there are several other probesets which also show spike-in behaviour in both spike-in datasets, as did several other authors [1, 2, 3]. Some of these are caused by different probesets mapping to the same transcript used in the spike-in experiment. However, some seem to be caused also by contamination of the spike-in groups. We calculated the correlation between the median-summarized spike-in signal and all individual probes, and calculated the number of probes per probeset that had a correlation over 0.75 with one of the spike-ins. If more than half of the probes of a probeset had such a high correlation, we removed the probeset from consideration, while if this was the case for all probes in a probeset we added the probeset as spike-in to the concentration group with the highest correlation. See Table 3 and 4 for the probesets that have been added. In Figure 1 we show the probe signals of the probesets that have been removed.

3 M-estimation

As earlier suggested in [4] we use M-estimation to estimate the used models. In normal least-squares optimization the square of the residual errors is minimized, i.e.

$$\min_{\phi} \sum_i (x_i - \theta)^2 \tag{1}$$

However, due to the squaring of the errors, strong outliers can have an adverse effect on the final solution. Instead one can use M-estimators, which are similar to least-squares for small outliers but reduce the weight of large outliers. In case of the Huber M-estimator, residuals beyond a certain threshold k only count linearly instead of squared to the error. Let $r_i = (x_i - \theta)$, then an Huber M-estimator weights residuals as:

$$\begin{cases} r_i^2 & \text{if} \quad |r_i| \leq k \\ 2k|r_i| - k^2 & \text{else} \end{cases} \tag{2}$$

Probesets added	Probesets removed
AFFX-DapX-5_at	203173_s_at
AFFX-DapX-M_at	204891_s_at
AFFX-LysX-5_at	208010_s_at
AFFX-LysX-M_at	
AFFX-PheX-5_at	
AFFX-PheX-M_at	
AFFX-ThrX-5_at	
AFFX-ThrX-M_at	
AFFX-r2-Bs-dap-5_at	
AFFX-r2-Bs-dap-M_at	
AFFX-r2-Bs-dap-3_at	
AFFX-r2-Bs-lys-5_at	
AFFX-r2-Bs-lys-M_at	
AFFX-r2-Bs-lys-3_at	
AFFX-r2-Bs-phe-5_at	
AFFX-r2-Bs-phe-M_at	
AFFX-r2-Bs-phe-3_at	
AFFX-r2-Bs-thr-3_s_at	
AFFX-r2-Bs-thr-M_s_at	
AFFX-r2-Bs-thr-5_s_at	
204890_s_at	
209374_s_at	

Table 3: Affymetrix HG_U133A spike-in experiment

Probesets added	Probesets removed
33818_at	546_at

Table 4: Affymetrix HG_U95A spike-in experiment

We used for the value k the value also used in [4], namely $k = 1.345\hat{s}$, where $\hat{s}$ is an estimate of scale defined as $\hat{s} = \text{median}(|r_i|)/0.6745$. The M-estimator can be solved by using iterative weighted least squares, adapting the weight $\mathbf{w}$ of the residuals within the least squares optimization:

$$\min_{r_i} \sum_i w_i r_i^2 \tag{3}$$

The weights are set in each iteration according to $\begin{cases} 1 & \text{if} \quad |r_i| \leq k \\ k/|r_i| & \text{else} \end{cases}$.

As stop condition we use the mean absolute difference between the residuals in the current and the last iteration. We stop the iterations when this falls below 0.01.

4 B-splines

In the sequence model we use B-splines to reduce the amount of parameters as well as overfitting. B-splines are modelled by using several control points p_i, i.e.

$$f(x) = \sum_i^P B_{i,d}(x) p_i \tag{4}$$

where P is the number of control points, d is the degree of the B-splines and $B_{i,d}(x)$ are the basis functions. Due to the additive model used, B-splines are relatively easy to use in a least-squares optimization problem.

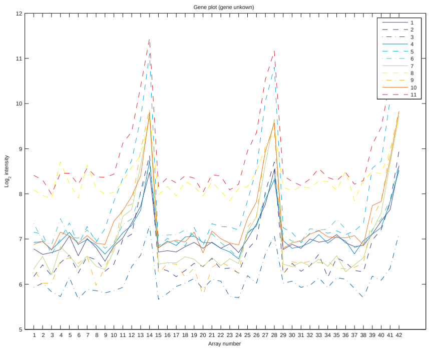

(a) HG_U133A: 203173_s_at

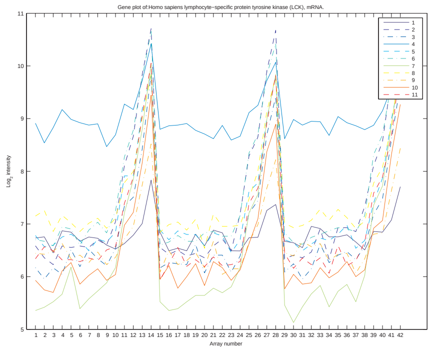

(b) HG_U133A: 204891_s_at

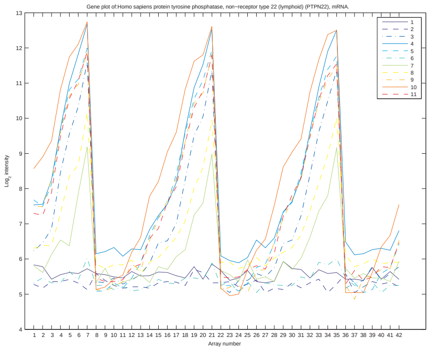

(c) HG_U133A: 208010_s_at

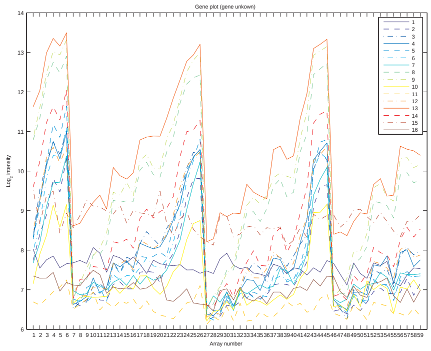

(d) HG_U133A: 546_at

Figure 1: Plots of the (unnormalized) probe signals of the removed 'spike-in' probesets. Each line represents a probe.

In our model, we use so-called clamped B-splines, where the first and last point clamp the spline. Given that the domain of x is $[0, 1]$, one can calculate the B-spline basis functions, by defining so-called knots along the domain of x. The number of knots has to be equal to $K = P + d + 1$. Furthermore, in order to generate clamped B-splines, the first and last knot has to be repreated $d+1$ times. The remaining knots are spaced equally over the domain of x. This results in the following knots: $k_0 = k_1 = ... = k_d = 0, k_{d+1}, ..., k_{K-d-1}, k_{K-d} = ... = k_K = 1$. Using these knots, the basis functions can be calculated using the following recursive formule:

$$\begin{aligned} B_{i,0}(x) &= \begin{cases} 1 & \text{if} \quad k_i \leq x < k_{i+1} \\ 0 & \text{else} \end{cases} \\ B_{i,d}(x) &= \frac{x-k_i}{k_{i+d}-k_i} B_{i,d-1}(x) + \frac{k_{i+d+1}-x}{k_{i+p+1}-k_{i+1}} B_{i+1,d-1}(x) \end{aligned} \tag{5}$$

This formulation can be adapted easily to other domains than $[0, 1]$, e.g. $[1, 25]$ for nucleotide position.

5 Amplification

It has been noticed before that probes toward the 5'end of the transcript have a lower expression than probes toward the 3'end of the transcript (e.g. [5, 6]). We found that this can not be seen as a 5'end degradation of the transcript, as probesets at different distances from the 5'end of the transcript (using the probe positions reported by Affymetrix), have the same average expression level (see Figure 2). Instead of interpreting this as an degradation effect progressing from the 5' end of the transcript, one can also explain it as an incomplete amplification effect. Amplification takes places from the 3'end of the transcript (as one can attach a general primer to the poly-A tail of the transcript).

5.1 Calculating distance to 3'end of transcript

Currently, Affymetrix probe positions are only given w.r.t. the 5' end of the transcript. To determine the positions w.r.t. to the poly-A tail of the transcript we downloaded the sequences of the targeted transcripts from Affymetrix. We used a simple heuristic to determine the location of the poly-A tail. From the position of the 3' end probe, we first search for the 'AATAAA' box to prevent false positive detection of the poly-A tail. Then, behind the 'AATAAA' box we search further for the poly-A tail using a heuristic regular expression: $[a]\{3, 1000\}.?[a]\{3, 1000\}.[0, 40]\$$. This finds a stretch of A nucleotides with one possible mismatch. At the very end of the mRNA molecule we allow for a stretch of mismatches. If no poly-A tail is found, we use the end of the transcript-sequence as the start of the poly-A tail. Using this, we obtain for every probe its position p_j w.r.t. the 3'end of the transcript.

Although this technique is reliable for most probes, using them during normalization showed that some poly-A tails are missed, presumably because there are other splice variants or because our method is not sophisticated enough. Furthermore, for some datasets (HG_U95A) the sequences did not cover the poly-A tail. For this reason we decided not to use the calculated distances in our normalization method. We used the described algorithm only to create Figure 4.

5.2 5'end bias can be explained by incomplete amplification

The distances of the probeset from the 3' end of the transcript are reported in Figure 4, showing that most probesets are situated closely to the 3' end of the transcript. For correct interpretation is has to be noted that probes are often positioned close together, in most cases covering a nucleotide distance less than 600 (see Figure 3). This explains why we do not see any differences in the expression level in Figure 2, as most probeset have the same relative distance w.r.t. 3' end of the transcript, independent of the distance from the 5' end of the transcript.

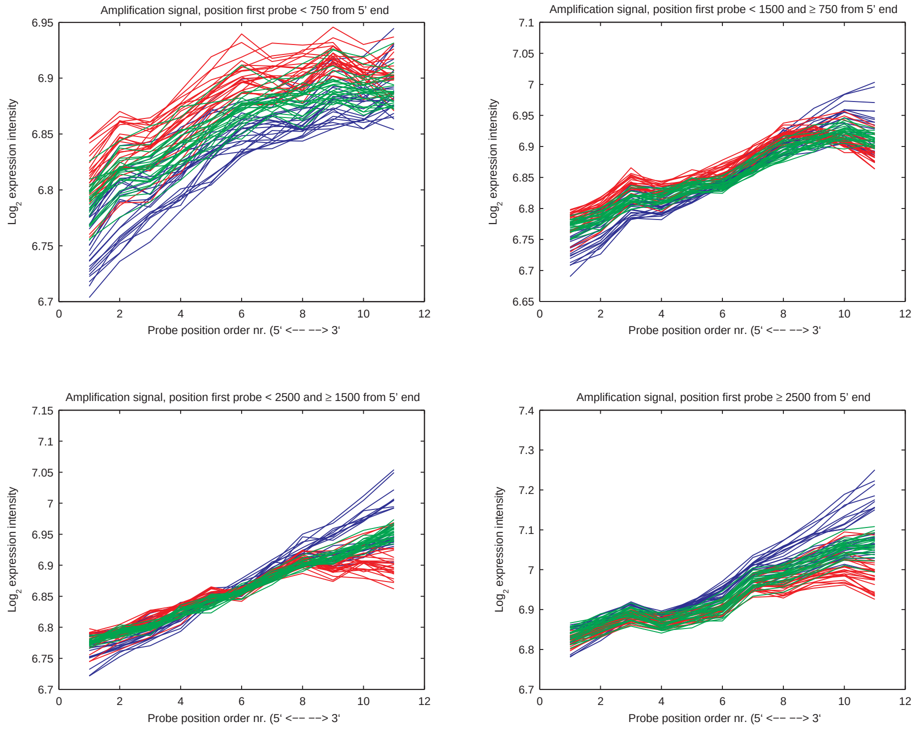

Figure 2: Amplification bias for the hMSC dataset. Probesets have been grouped into four subsets based on the distance of their first probe to the 5'end of the transcript. For each subset, we report the median signal of the different probes in the probesets, ordered on position from the 5' end of the transcript. Most probesets contain 11 probes. Probesets with more or less probes are interpolated. The different lines in the plots are the different arrays, while the different colors indicate the different batches. The number of probesets in each subplot is approximately equal, each reporting on more than 5000 probesets (on a total of 22277 probesets).

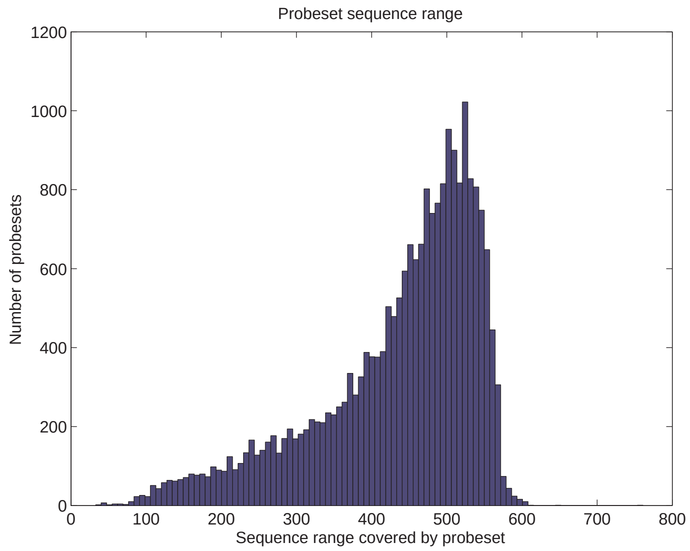

Figure 3: The sequence range covered by the probes in a probeset (HG_U133A 2.0 platform)

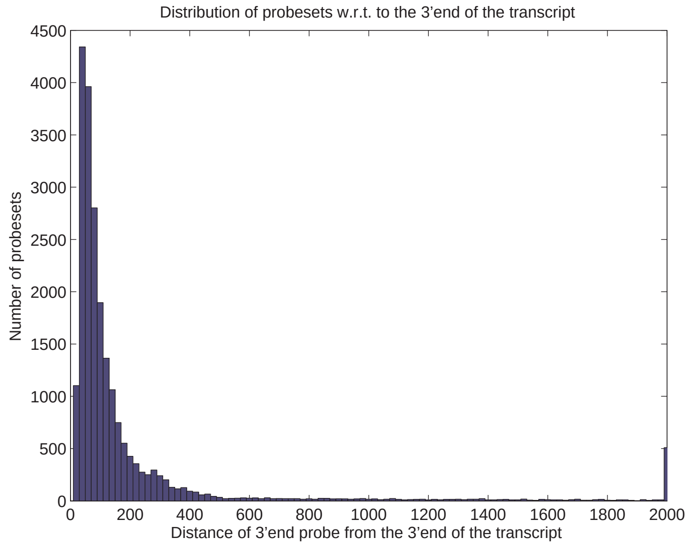

Figure 4: The distribution of the distance between the 3' end of the probeset and the 3' end of the transcript. (HG_U133A 2.0 platform)

Method name	Normalization residual (sum)	Residual array 8	Residual array 14
No amplification correction	1.0794 * 10e6	3.8349 * 10e4	4.2113 * 10e4
Linear position-based model	1.0676 * 10e6	3.7271 * 10e4	4.0182 * 10e4
Sequence-based model	1.0464 * 10e6	3.5926 * 10e4	3.8202 * 10e4

Table 5: Normalization residuals for the hMSC dataset with and without removing amplification differences between the arrays. Both the sum over all arrays, and some arrays particularly affected by amplification issues are shown.

5.3 Sequence affects amplification effect

Although Figure 2 seems to imply that a linear, position-based model should work well for modelling amplification, we found this not to be the case. Although such a model does fit some of the amplification effect a large part still remains after correction. Based on our results, we think that although the average in the graph shows a direct linear relation, this does not have to be the case for individual probe sets. One can easily imagine that these amplification effects are influenced by the sequence. The sequence model we used is described in the paper. Here we report residual norms after using the different models (Table 5). As can be seen, the sequence-based model reduces the residual norm significantly more than the linear-position based model, indicating that sequence indeed affects the amplification effect. An F-test on these results shows that this improvement is highly significant ($pvalue = 4.7e - 15$).

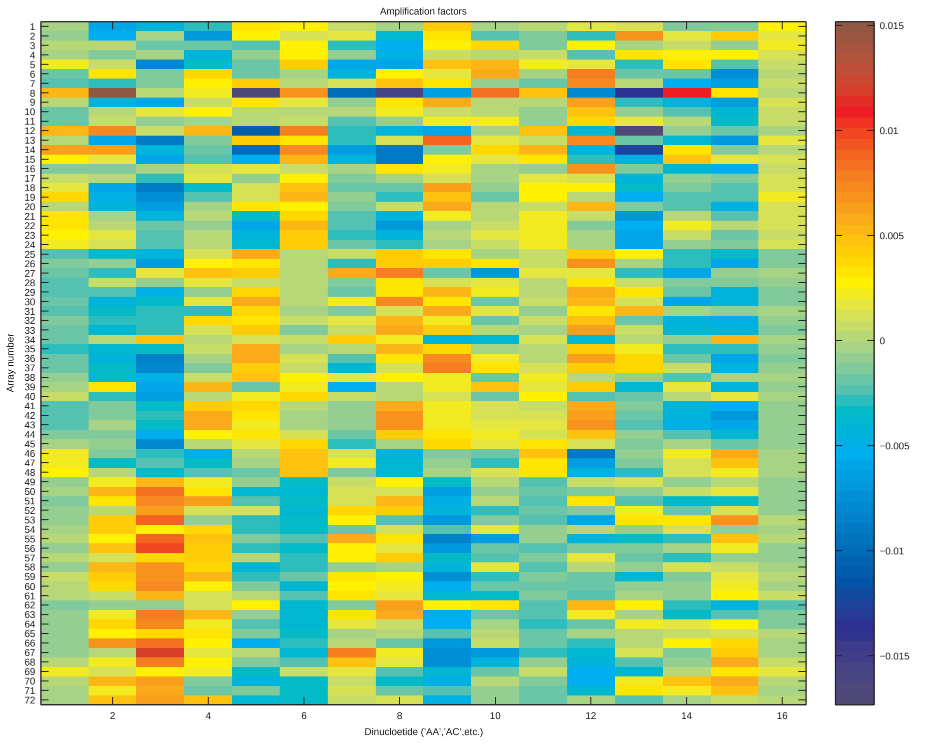

Figure 5: Amplification dinucleotide weights for the hMSC dataset. Each row represents the weights of an array, every column a certain dinucleotide. Rows are ordered on batch (batch 1: array 1-24, batch 2: array 25-48, batch 3: 49-72).

6 Array location effect

We found that the array location effect seems not to affect the optical background O. This is illustrated by Figures 7 and 8. Let the residue be described as $r_{ij} = \log_2 [s_{ij}] - \log_2 \left[\text{median}_i (s)_j\right]$ (signals normalized using quantile-quantile normalization). Then, for the probes in the top left part of the array shown in Figure 7, we show the residues in Figure 8. This shows that the probes with the lowest expression values are not influenced by the image effect.

To determine if this was the case for all arrays, we optimized the optical factor to reduce the residue values. We compared the result with the optical scaling factors determined using the minimum PM value, as suggested in [7] based on experiments. We find that these values are approximately comparable (see Figure 9), suggesting that indeed the optical component of the signal is not influenced by array location effects.

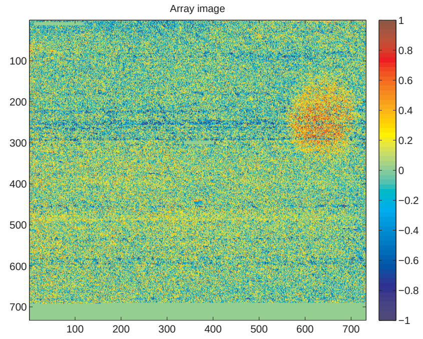

(a) hMSC dataset, before normalization

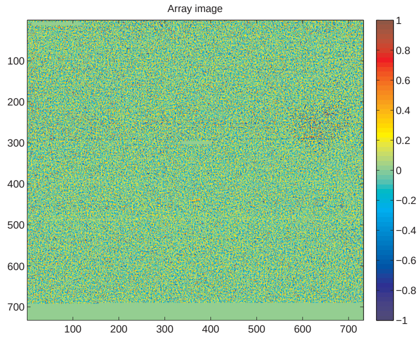

(b) hMSC dataset, after normalization

Figure 6: Array image of array 1 of the hMSC dataset, showing the $\log_2$ difference of each probe w.r.t. to the $\log_2$ median signal before and after normalization.

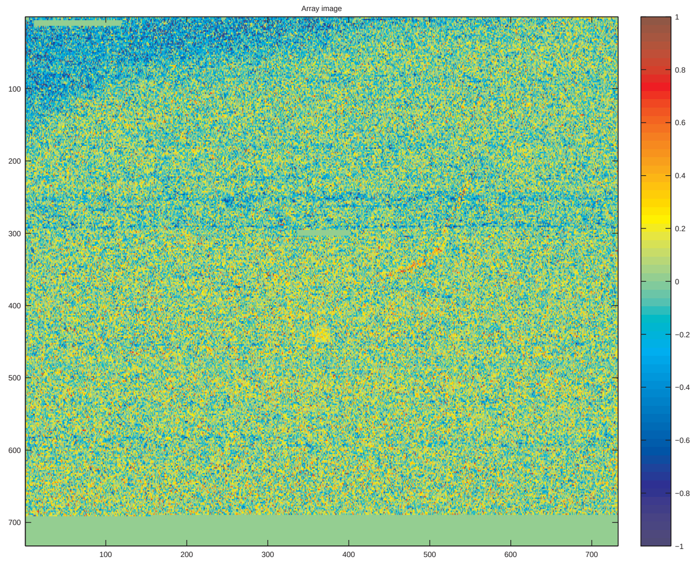

Figure 7: Array image of array 4 of the hMSC dataset, showing the $\log_2$ difference of each probe w.r.t. to the $\log_2$ median signal.

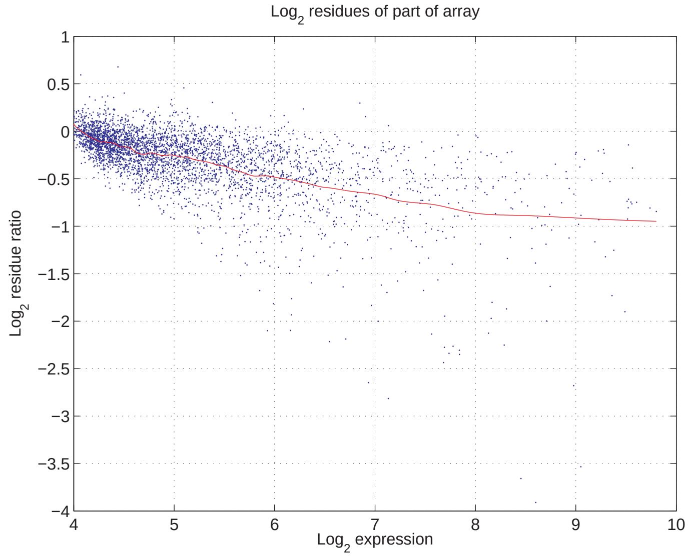

Figure 8: The image correction factors obtained for the probes in the top left part of Figure 7 using a median box filter of size 9x9.

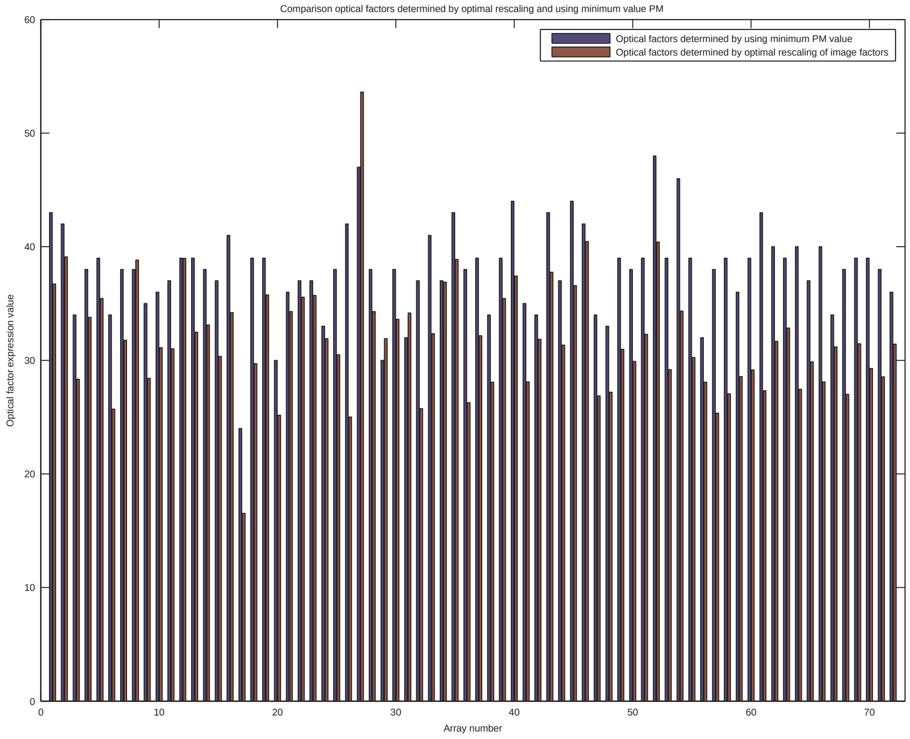

Figure 9: Comparison optical factors determined by rescaling the image factors and using the minimum PM value.

Method name	r^2	slope
MAS 5.0	0.7941	0.6939
PLIER	0.9209	0.7138
VSN	1.1121	0.5088
PDNN	**0.4460**	0.3252
RMA_NBG	1.2143	0.4741
RMA	0.7582	0.6086
GCRMA	1.3398	**0.8416**
RDN	1.1754	0.4881
RDN [no backscaling]	0.8003	0.5938
RDN [$\eta = 1$, no backscaling]	0.6144	0.6722

Table 6: Residual and slope values for expression signal versus spike-in concentration for the HG_U95A spike-in dataset.

Method name	r^2	slope
MAS 5.0	0.5202	0.7655
PLIER	**0.2065**	0.8273
VSN	3.4808	0.5303
PDNN	1.4350	0.3906
RMA_NBG	3.5121	0.5289
RMA	2.6345	0.6760
GCRMA	4.0704	**0.9735**
RDN	3.3760	0.5459
RDN [no backscaling]	2.0138	0.6704
RDN [$\eta = 1$, no backscaling]	0.5424	0.7999

Table 7: Residual and slope values for expression signal versus spike-in concentration for the HG_U133A spike-in dataset.

7 Signal bias

For each line in Figure 6c and 6d in the paper, we calculated the summed r^2 measure, as well as the slope of the line (excluding the first part of the line, from expression 0 to 0.13 as this is not a 2-fold expression step). The results are in Tables 6 and 7.

References

[1] M. McGee and Z. Chen, "New spiked-in probe sets for the affymetrix hgu-133a latin square experiment," in *COBRA Preprint Series 2006*, 2006.

[2] C. Wu, R. Carta, and L. Zhang, "Sequence dependence of cross-hybridization on short oligo microarrays," *Nucleic Acids Research*, vol. 33, no. 9, 2005.

[3] W. Hsieh, T. Chu, R. Wolfinger, *et al.*, "Who are those strangers in the latin square?," *Methods of Microarray Data Analysis III: Papers from CAMDA'02*, 2003.

[4] B. Bolstad, *Low-level Analysis of High-density Oligonucleotide Array Data: Background, Normalization and Summarization.* PhD thesis, University of California, 2004.

[5] Affymetrix, "Affymetrix microarray suite user guide (version 5)," tech. rep., Affymetrix Santa Clara, 2002.

[6] L. Gautier, L. Cope, B. Bolstad, and R. Irizarry, "affy–analysis of affymetrix genechip data at the probe level," *Bioinformatics*, vol. 20, no. 3, p. 307, 2004.

[7] Z. Wu and R. A. Irizarry, "Stochastic models inspired by hybridization theory for short oligonucleotide arrays," *Journal of Computational Biology*, vol. 12, no. 6, p. 882, 2005.
